# Renoprotective Effect of Pitavastatin against TAA-Induced Renal Injury: Involvement of the miR-93/PTEN/AKT/mTOR Pathway

**DOI:** 10.1155/2024/6681873

**Published:** 2024-01-23

**Authors:** Marawan A. Elbaset, Bassim M. S. A. Mohamed, Passant E. Moustafa, Tuba Esatbeyoglu, Sherif M. Afifi, Alyaa F. Hessin, Sahar S. Abdelrahman, Hany M. Fayed

**Affiliations:** ^1^Department of Pharmacology, Medical Research and Clinical Studies Institute, National Research Centre, Giza, Egypt; ^2^Department of Molecular Food Chemistry and Food Development, Institute of Food Science and Human Nutrition, Gottfried Wilhelm Leibniz University Hannover, Am Kleinen Felde 30, Hannover 30167, Germany; ^3^Pharmacognosy Department, Faculty of Pharmacy, University of Sadat City, Sadat City 32897, Egypt; ^4^Department of Pathology, Faculty of Veterinary Medicine, Cairo University, Giza, Egypt

## Abstract

This research investigated if pitavastatin (Pita) might protect rats' kidneys against thioacetamide (TAA). By altering the PTEN/AKT/mTOR pathway, pitavastatin may boost kidney antioxidant capacity and minimize oxidative damage. Statins have several benefits, including antioxidant and anti-inflammatory characteristics. The principal hypothesis of this study was that Pita can regulate the miR-93/PTEN/AKT/mTOR pathways, which is thought to be responsible for its renoprotective effects. The experiment divided male rats into four groups. Group 1 included untreated rats as the control. Group 2 included rats which received TAA (100 mg/kg intraperitoneally thrice a week for two weeks) to destroy their kidneys. Groups 3 and 4 included rats which received Pita orally at 0.4 and 0.8 mg/kg for 14 days after TAA injections. Renal injury increased BUN, creatinine, and MDA levels and decreased glutathione (GSH) levels. Pitavastatin prevented these alterations. TAA decreased PTEN and increased miR-93, Akt, *p*-Akt, mTOR, and Stat3 in the kidneys. Pitavastatin also regulated the associated culprit pathway, miR-93/PTEN/Akt/mTOR. In addition, TAA induced adverse effects on the kidney tissue, which were significantly ameliorated by pitavastatin treatment. The findings suggest that pitavastatin can attenuate renal injury, likely by regulating the miR-93/PTEN/Akt/mTOR pathway. This modulation of the pathway appears to contribute to the protective effects of pitavastatin against TAA-induced renal injury, adding to the growing evidence of the pleiotropic benefits of statins in renal health.

## 1. Introduction

Acute kidney injury (AKI) is a clinical illness characterized by abruptly impaired renal function, retention of metabolites, and abnormalities of the water, electrolyte, and acid-base balance [1]. AKI is a severe condition that causes over 2 million fatalities globally each year and is linked to higher morbidity and mortality rates [2]. Prior research has shown that patients with AKI frequently experience incomplete recovery of their renal function. In addition, AKI is a substantial risk factor for the development of both chronic kidney disease (CKD) and end-stage renal disease (ESRD) [3]. Inflammation and oxidative stress are intricately interconnected, leading to a detrimental cycle. Oxidative stress initiates the process of inflammation, which, in response, further triggers oxidative stress by generating reactive oxygen species (ROS). These harmful occurrences collectively contribute to tissue damage by inducing necrosis, apoptosis, and fibrosis [4]. AKI lacks specific treatments beyond standard supportive care and renal replacement therapy, which significantly contributes to the unfavorable short-term outlook experienced by AKI patients [4, 5]. Despite substantial research on AKI, the molecular pathways underlying renal damage have not yet been thoroughly explored. In order to treat or stop the progression of AKI to CKD, new medications or mechanisms must be developed. Despite extensive research on AKI, there remains a lack of comprehensive understanding regarding the molecular processes underlying renal injury. Consequently, it is essential to explore new medications or mechanisms to effectively manage or prevent the progression of AKI to CKD [6].

MicroRNAs (miRs) are short RNA molecules, typically consisting of 18–25 nucleotides, which control protein translation by binding to complementary regions of mRNA. This binding typically occurs in the 3′ untranslated region of the mRNA [7]. In studies involving preclinical AKI models, numerous miR variations have demonstrated promising therapeutic capabilities [8]. For instance, in cases of experimental acute kidney injury (AKI) caused by ischemia-reperfusion (I/R), administering miR-17-5p mimic via intravenous injection has been shown to hinder histologic ischemic damage. It also leads to a decrease in blood urea nitrogen and serum creatinine levels.

In addition, when miR-21 is overexpressed in mice, it offers protection against ischemic kidney injury by reducing apoptosis [9–11]. In addition, Liao and colleagues [12] provided evidence that miR-140-5p enhanced the recovery from cisplatin-induced AKI in mice. This effect was achieved by reducing oxidative stress through the activation of the Nrf2/ARE pathway. Moreover, recent research has unveiled the protective properties of miRNAs in different renal cell types, as they effectively inhibit apoptosis and inflammatory responses [13, 14].

Phosphatase and tensin homolog (PTEN), located on chromosome 10, acts as a tumor suppressor by exhibiting the bispecific phosphatase activity. This activity leads to the dephosphorylation of PI3P, converting it into PI2P. Consequently, the phosphatidylinositol 3-kinase (PI3K)/Akt signaling pathway is deactivated [15]. PI3K becomes activated, leading to the phosphorylation of PIP2 and its conversion into PIP3. Consequently, Akt is activated by PIP3 [16]. Furthermore, there is a notable rise in the levels of PTEN, leading to the suppression of the Akt/mTOR signaling pathway [17]. Akt stimulates mTOR, leading to the initiation of downstream protein translation through the phosphorylation of 4EBP1 and P70S6K [18]. All renal tissues express PTEN. However, renal tubular cells are where it is most abundant [19].

Statins are a traditional medication utilized to address hyperlipidemia in obese and diabetic patients. Their mode of action involves the inhibition of the hydroxyl-3-methylglutaryl-coenzyme A (HMG-CoA) reductase enzyme, which not only leads to a direct reduction in endogenous cholesterol production but also exhibits additional beneficial effects known as pleiotropic effects, as demonstrated in both laboratory experiments (*in vitro*) and studies involving living organisms (*in vivo*) [20]. In addition to reducing oxidative stress, inflammation, and apoptosis, in streptozotocin-induced type 1 diabetes models in rats, statins may improve pancreatic and renal function [21]. In addition, the beneficial impacts of statins on kidney dysfunction were observed in rats with diet-induced obesity and patients diagnosed with type 2 diabetes (T2DM) [22–26]. The kidney-protective benefits of atorvastatin, simvastatin, and rosuvastatin have been proven [27]. Statins demonstrate advantageous impacts in various animal model kidney diseases [28]. Despite numerous research papers focusing on statins' impact on AKI, this study had a distinct objective of examining pitavastatin (Pita)'s potential in mitigating thioacetamide- (TAA-) induced renal injury in rats. The main hypothesis of this investigation was that Pita's renoprotective properties are attributed to its regulation of miR-93/PTEN/AKT/mTOR pathways.

## 2. Materials and Methods

### 2.1. Animals

Twenty-four adult male Wistar rats weighing 150–200 g and five months old were acquired from the “Animal House Colony at the National Research Centre (NRC, Cairo, Egypt).” Rats were housed at ambient temperature (25°C) and on a 12-hour light/dark cycle. All operations and tests with permission number “1041112022” have been authorized by the National Research Center's Medical Research Ethics Committee. The report of the study follows the ARRIVE principles in accordance with ARRIVE guidelines.

### 2.2. Chemicals

From Sigma-Aldrich Co. (St. Louis, MO, USA), thioacetamide (TAA) was purchased. From Western pharmaceutical industries, in Egypt, pitavastatin was acquired. Every other chemical that was used in the experiments was of the highest purity and analytical grade. Freshly suspended Pita was orally administered at a dose of 0.4 mg/kg and 0.8 mg/kg in a 1% Tween 80 solution [29].

### 2.3. Experimental Design

The rats were divided into four groups (6 rats/group). Rats in group 1 (negative control group) were given an intraperitoneal (ip) injection of saline three times a week for two consecutive weeks. Rats in group 2 (TAA group) received an intraperitoneal (ip) injection of TAA (100 mg/kg) three times a week for two consecutive weeks to cause renal injury [30]. Rats in groups 3 and 4 were given Pita orally (0.4 and 0.8 mg/kg) [31] every day for two weeks following TAA injections.

### 2.4. Preparation of Serum Samples

After completing the experiment, the rats were deprived of food overnight. Their blood was then drawn, and the serum was extracted by spinning the samples for five minutes at 3000 rpm and 4°C. The obtained serum was then stored at −80°C for later use in measuring blood urea nitrogen (BUN) and creatinine levels.

### 2.5. Collecting Renal Tissues

A cold saline solution was used to rinse the kidneys. They were then split into three portions. To enable mRNA extraction and the evaluation of miRNA 93 and AKT gene expression, the first portion was promptly frozen in liquid nitrogen and kept at a temperature of −80°C. After homogenizing the second portion with 0.5% potassium chloride (1 mL for every 100 mg of tissue), it was centrifuged for 10 min at 4°C and 3000 rpm. The resultant supernatant was separated and stored at 80°C to measure renal glutathione (GSH) and malondialdehyde (MDA) using colorimetric kits, as well as AKT, *p*-AKT, mTOR, *p*-mTOR, and PTEN using ELISA. At the same time, the second portion was utilized to estimate the renal content of miRNA 93 and the AKT RNA content. Finally, the last part was fixed in 10% buffered formalin for subsequent histopathological and immune-histochemical investigations.

### 2.6. Renal Function Tests

To assess the renal function, we measured serum BUN and creatinine levels using commercially available kits. BUN levels were determined colorimetrically with the BioVision kit (Catalog# K375-100, Milpitas Boulevard, Milpitas, USA). The principle of this is a kit comprised of the hydrolysis of urea by urease to produce ammonia and CO_2_. The ammonia that is produced then combines with hypochlorite and a phenolic chromogen to generate a complex that is green in color. The color intensity that develops is closely correlated with the urea content of the sample and vice versa. Besides, creatinine levels were measured colorimetrically using the BioVision kit (Catalog# E4370-100, Milpitas Boulevard, Milpitas, USA). The principle of this kit comprised the modified kinetic Jaffe methodology for the determination of creatinine involving a protein-free filtrate and a reaction with picric acid in an alkaline solution, a surfactant, and other ingredients to minimize protein and carbohydrate interferences [32]. The assays were performed following the manufacturer's instructions.

### 2.7. Renal Oxidative Stress Markers

For the evaluation of renal oxidative stress, we measured MDA levels and the GSH content. The MDA level was assessed colorimetrically with the BioVision kit (Catalog# K739-100, Milpitas Boulevard, Milpitas, USA) that is based on the reaction of MDA in the sample with thiobarbituric acid (TBA) to generate the MDA-TBA adduct which can be easily quantified colorimetrically (OD 532 nm). The GSH content was determined colorimetrically using the BioVision kit (Catalog# K464-100, Milpitas Boulevard, Milpitas, USA) based on an enzymatic cycling method in the presence of GSH and a chromophore. The reduction of the chromophore produces a stable product, which can be followed kinetically at 450 nm. The procedures were conducted in accordance with the manufacturer's instructions to ensure accuracy and reliability.

### 2.8. Detection of mRNA Expression of miRNA 93 and Akt in Renal Tissues via Real-Time qRT-PCR

Thermo Fisher Scientific, Waltham, Massachusetts, USA, supplied the SuperScript IV One-Step RT-PCR kit for reverse transcription of extracted RNA and PCR. The following thermal profile was created using a StepOne instrument (Applied Biosystem, USA): 10 minutes at 45°C for reverse transcription, 2 minutes at 98°C for RT inactivation, and initial denaturation by 40 cycles of 10 s at 98°C, 10 s at 55°C, and 30 s at 72°C for the amplification step. Data were expressed as cycle threshold (Ct) for the target genes and housekeeping gene following the RT-PCR run. Using the mean critical threshold (CT) expression levels of the housekeeping gene GAPDH, the target genes' expression was normalized for variation using the ΔΔCt method. Each target gene's relative quantitation (RQ) is determined using the 2^−∆∆Ct^ method of calculation. The AKT gene's primer sequence was 5′-CGGATACCATGAACGACGTAG-3′ and 5′-GCAGGCAGCGGATGATAAAG-3′; the GenBank accession number for this sequence is NM_033230.2 which was normalized versus the GAPDH as a housekeeping gene; forward 5′-TGGCCTTCCGTGTTCCTAC-3′ and reverse 5′-GAGTTGCTGTTGAAGTCGCA-3′ (GenBank accession number is XM_036165840.1). The miRNA 93 gene's primer sequence is as follows: forward 5′-CAAAGTGCTGTTCGTGCAGGTAG-3′; reverse 5′-AACGCTTCACGAATTTGCGT-3′; normalization was performed against housekeeping gene U6 forward 5′- CTCGCTTCGGCAGCACA-3′, and reverse 5′-AACGCTTCACGAATTTGCGT-3′ (GenBank accession number is K00784.1).

### 2.9. ELISA Assay

The protein concentrations of *p*-AKT were measured using commercially available ELISA kits (Catalog# PEL-AKT-S473-T, Ray Biotech, Norcross, USA), mTOR or *p*-mTOR (Catalog# SL1350Ra and SL5329R SunLong Biotech Co., Zhejiang, China), and AKT or PTEN (Catalog# MBS3807575 and MBS452729, MyBioSource, San Diego, USA) in renal tissue homogenate. Each ELISA kit's manufacturer's instructions were followed.

### 2.10. Histopathological Examination

The renal tissues were fixed for 24 h in 10% buffered formalin. After that, it was dried in various alcohol concentrations, cleaned in xylene, and finally embedded in paraffin wax. The paraffin slices (4 mm) were stained with hematoxylin and eosin (H&E) stain. The cells were inspected under a light microscope by a blinded pathologist to prevent prejudice.

### 2.11. Immune-Histochemical Examination of STAT3 Expression

The other paraffin section from each group was used for immunohistochemical detection of the expression of Stat3 in various experimental groups using avidin-biotin-peroxidase according to the method described in [33]. Kidney slices were treated with monoclonal antibodies for Stat3 (Abcam, Cambridge, MA, USA) at a dilution of 1 : 200 and (Vactastain ABC peroxidase kit, Vector Laboratories, Newark, USA) for the aim of identifying antigen-antibody complexes. Each marker's expression was visualized using chromagen 3,3-diaminobenzidine tetrahydrochloride (DAB, Sigma Chemical Co.). The positive brown region of each marker's expression was assessed using seven high-power microscopic fields and image analysis software (ImageJ, 1.46a, NIH, Bethesda, USA).

### 2.12. Statistical Analysis

The outcomes are represented as the “means ± S.E.”. Data were processed by “one-way ANOVA followed by the Tukey–Kramer post hoc test.” “GraphPad Prism software (version 9, USA)” was used to conduct the statistical analysis and create the graphs shown. The significance level was set to “*p* < 0.05” for all statistical tests [34].

## 3. Results

### 3.1. Effect of Pitavastatin on Renal Function Tests

**Figure 1:**
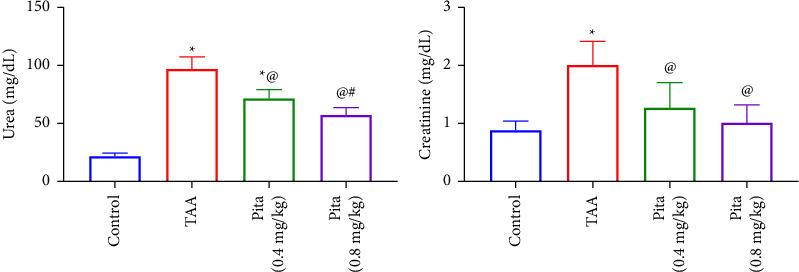
Effect of pitavastatin on serum renal function: (a) urea and (b) creatinine. A bar shows the mean and standard error of six rats: ^*∗*^compared to the normal control group, ^@^to the TAA group, and ^#^to pita (0.4 mg/kg) at *p* < 0.05. Pita: pitavastatin and TAA: thioacetamide.

The serum levels of creatinine and BUN significantly increased after the injection of TAA. Pita administration successfully reduced serum creatinine and BUN levels in both treatment groups, as indicated in Figure 1.

### 3.2. Effect of Pitavastatin on the Oxidative Stress Status

**Figure 2:**
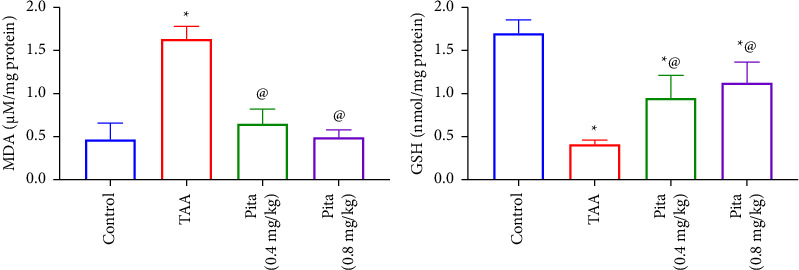
Effect of pitavastatin on renal (a) MDA and (b) GSH. A bar shows the mean and standard error of six rats: compared ^*∗*^to the normal control group and ^@^to the TAA group at *p* < 0.05. Pita: pitavastatin and TAA: thioacetamide.

In the TAA group, compared to the control group, there was a significantly higher level of renal MDA (*p* < 0.05) and a significantly lower level of renal GSH (*p* < 0.05). As indicated in Figure 2, Pita treatment successfully reduced the renal MDA content in both treated groups (*p* < 0.05) and enhanced the GSH content when compared to the TAA group (*p* < 0.05).

### 3.3. Effect of Pitavastatin on the PTEN/AKT/mTOR Pathway

**Figure 3:**
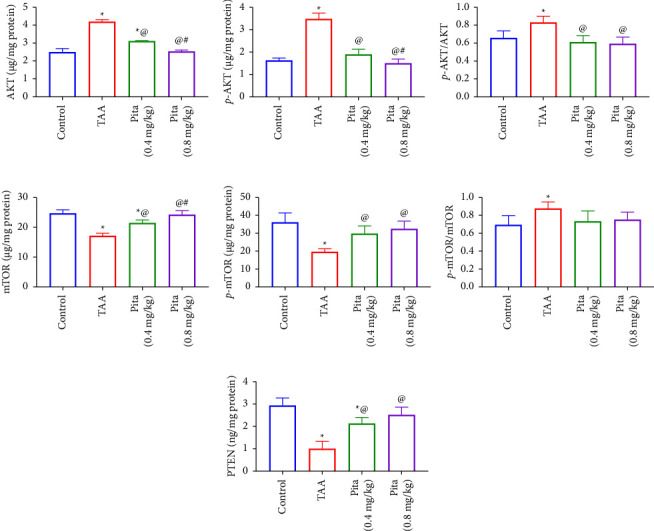
Effect of pitavastatin on (a) AKT, (b) *p*-AKT, (c) *p*-AKT/AKT, (d) mTOR, (e) *p*-mTOR, (f) *p*-mTOR/m-TOR, and (g) PTEN. A bar shows the mean and standard error of six rats: ^*∗*^compared to the normal control group, ^@^to the TAA group, and ^#^to pita (0.4 mg/kg) at *p* < 0.05. Pita: pitavastatin and TAA: thioacetamide.

The level of PTEN and the downstream variables AKT, *p*-AKT, mTOR, and *p*-mTOR were examined in order to clarify further the mechanism underlying the protective effect of Pita against renal injury because these factors are crucial in the development of renal injury. PTEN levels were significantly decreased, and AKT, *p*-AKT, mTOR, and *p*-mTOR levels were significantly increased by TAA-induced renal injury compared to the control group (*p* < 0.05). In contrast, PTEN levels were significantly decreased, and AKT, *p*-AKT, mTOR, and *p*-mTOR levels were significantly increased by the Pita treatment groups (*p* < 0.05, Figures 3(a)–3(e)).

### 3.4. Effect of Pitavastatin on Renal miR-93 and AKT Gene Expression

**Figure 4:**
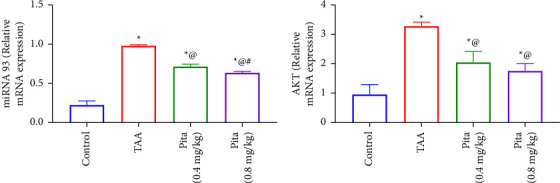
Effect of pitavastatin on renal gene expression of (a) miR-93 and (b) AKT. A bar shows the mean and standard error of six rats: ^*∗*^compared to the normal control group, ^@^to the TAA group, and ^#^to pita (0.4 mg/kg) at *p* < 0.05. Pita: pitavastatin and TAA: thioacetamide.

In the TAA model group (*p* < 0.05), as compared to the control group, real-time qRT-PCR analysis revealed a significantly increased expression of miR-93 and AKT. Comparing the gene expression of miR-93 and AKT in both Pita-treated groups to TAA-renal damaged rats, Figure 4 demonstrates a significant decrease in both genes' expression (*p* < 0.05).

### 3.5. Histopathological Findings

**Figure 5:**
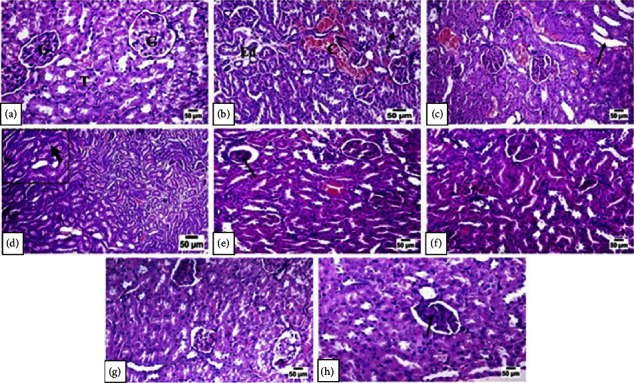
Effect of *p* on histopathology findings. Normal control (a), TAA group (b–d), low dose of pitavastatin-treated groups (e–f), and high dose of the pitavastatin-treated group (g–h).

Pita markedly prevented the adverse effects of TAA on the kidney tissue. Normal histological structures of cortical and medullary elements, as well as the renal pelvis, were clearly noticed under light microscopic examination of the kidney tissues of control rats (Figure 5(a)). Whilst TAA administration had an adverse effect on kidney tissue, particularly in the cortex, which showed variation in the tinctorial intensity of the renal tubules with marked dilatation and congestion of the interstitial blood vessels with interstitial edema and few pockets of hemorrhages (Figure 5(b)). The renal tubules showed epithelial lining swelling, degeneration, some pyknotic nuclei, necrosis, and sloughing of a few cells. Other tubules were cystically dilated with an attenuated epithelium (Figure 5(c)). Some cellular debris was observed in the lumen of a few tubules. Occasional granular casts were present in medullary collecting ducts and tubules (Figure 5(d)). Most of the glomeruli showed membrane thickening of the glomerular tuft with few cellular infiltrates and few others appeared with atrophied tufts. Daily treatment with Pita in rats for two weeks prior to being administrated TAA showed marked protection of their kidney tissues against the harmful effect of TAA, particularly in the high-dose administrated rats. Rats administrated Pita at a low dose (Figures 5(e) and 5(f)) showed mild degenerative changes of the renal tubular epithelium with scares glomeruli appearing with atrophied tuft. While at the high dose of Pita (Figures 5(g) and 5(h)), mild degenerative changes were noticed in some tubular epithelium with the appearance of scattered foci of regenerated tubules.

Pita reduced TAA's kidney damage. Under light microscopy, the kidney tissues of control rats showed the normal histological structure of cortical, medullary, and renal pelvic components (Figure 5(a)). TAA treatment damaged the kidney tissue, notably in the cortex, which demonstrated variance in renal tubule tinctorial intensity, significant dilatation and congestion of interstitial blood vessels, interstitial edema, and rare hemorrhages (Figure 5(b)). The renal tubules revealed epithelial lining enlargement, degeneration, some pyknotic nuclei, necrosis, and sloughing of a few cells. Other cystically dilated tubules have attenuated epithelium (Figure 5(c)). Few tubules had cellular debris. Medullary collecting ducts and tubules sometimes have granular deposits (Figure 5(d)). Most glomeruli had membrane thickening and minimal cellular infiltrates, whereas a few had atrophied tufts. Pita given daily to rats for two weeks before TAA administration protected their kidney tissues, especially in high-dose animals. Low-dose Pita-treated rats (Figures 5(e) and 5(f)) demonstrated modest renal tubular epithelial degeneration with scarred glomeruli and atrophied tuft. At high Pita doses (Figures 5(g) and 5(h)), some tubular epithelium showed modest degeneration and scattered foci of regenerated tubules.

### 3.6. Immunohistochemistry Findings

**Figure 6:**
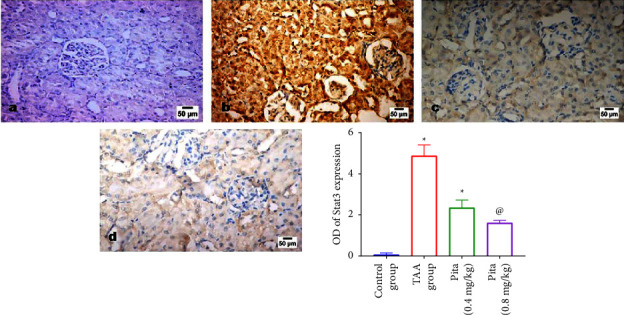
Effect of pitavastatin on Stat3 expression in kidney tissues. (a) Normal group, (b) TAA group, (c) low dose of Pita, and (d) high dose of Pita. A bar shows the mean and standard error of six rats: compared ^*∗*^to the normal control group and ^@^to the TAA group at *p* < 0.05. Pita: pitavastatin and TAA: thioacetamide.

A remarkable increased expression of Stat3 was noticed in the kidney tissues of the rats' group administrated TAA (Figure 6(b)) compared to the control group (Figure 6(a)). Meanwhile, Pita (0.4 and 0.8 mg/kg) treatment following TAA administration displayed a dose-related reduced Stat3 expression compared to the TAA group (Figures 6(c) and 6(d)). The quantitative analysis of the positive brown color of Stat3, displayed as a staining score, demonstrated that the TAA rats significantly (*p* < 0.05) overexpressed Stat3 in comparison to the other treated groups.

TAA-treated rats had significantly higher Stat3 expression in their renal tissues (Figure 6(b)) than controls (Figure 6(a)). Pita (0.4 and 0.8 mg/kg) therapy after TAA lowered Stat3 expression compared to the TAA group (Figures 6(c) and 6(d)). TAA rats overexpressed Stat3 based on the Stat3 staining score.

## 4. Discussion

Statins work by blocking cholesterol synthesis, leading to a reduction in blood lipids. The recent focus on the pleiotropic effects of statins arises from numerous clinical and preclinical investigations that highlight the additional benefits of statins beyond cholesterol control. These studies reveal advantages that are not directly related to cholesterol levels [35]. Pita stands as an innovative statin with remarkable pharmacokinetic and pharmacodynamic qualities. Unlike other statins, it undergoes minimal metabolism by CYP3A4 (cytochrome P450), reducing the likelihood of drug interactions with substances metabolized by this enzyme. Additional research has revealed several advantageous attributes of Pita, including safeguarding endothelial function and suppressing vascular inflammation and oxidative stress [36]. Thioacetamide (TAA), an organosulfur compound, is commonly used as a fungicide [37]. TAA is converted *in vivo* to the free radical derivatives TAA sulfoxide and TAA-S, S-dioxide. This causes enhanced lipid peroxidation, which leads to the generation of reactive oxygen species (ROS), which causes damage to the liver [38–41]. The metabolites produced can affect amine lipids and proteins, increasing systemic oxidative stress, releasing cytokines, and altering kidney function since they are dispersed laterally throughout various organs, including the liver, kidney, adrenals, bone marrow, and other tissues [42–44].

While previous studies have shown the nephroprotective effects of statins, including pitavastatin [45–51], the specific involvement of the miR-93/PTEN/Akt/mTOR pathway in mediating these effects has not been elucidated *in vivo* in the context of xenobiotic-induced kidney injury. Our study is the first to demonstrate that pitavastatin attenuates TAA-induced kidney damage in rats by regulating this essential signaling cascade. We used the TAA model because it is a well-established approach for studying mechanisms of kidney injury due to oxidative stress and xenobiotic toxicity.

Our findings significantly advance understanding of the mechanisms underlying statins' pleiotropic benefits in the kidney. We show for the first time the integral role of miR-93 and PTEN regulation in activating downstream Akt/mTOR signaling to mitigate renal injury. These novel insights expand the evidence for the multifaceted protective properties of statins and support their potential therapeutic use to preserve kidney function in contexts of toxicant exposure or oxidative damage. Our study also suggests that the miR-93/PTEN/Akt/mTOR axis could be a promising target for future nephroprotective strategies.

In our research, we observed notable disruption in kidney function among rats treated with TAA, as indicated by increased levels of BUN and creatinine in the bloodstream. Prior studies had already indicated the potential impact of TAA on measures of the kidney function [52]. Pita normalized the levels of BUN and creatinine in a dose-dependent manner. Our findings are in accordance with [53]. The histopathological examination revealed considerable tissue damage and loss of structure in the kidneys of the group treated with TAA. However, the group treated with Pita showed a noteworthy decrease in tissue damage, along with improved preservation of cellular nuclei and organelles.

In alignment with prior research, it was observed that alongside abnormal kidney function and histological changes, there was an elevation in MDA levels and a decrease in the antioxidant content (specifically GSH). These results correspond with earlier studies [54, 55]. TAA can cause a significant amount of ROS to be produced, which can hinder the antioxidant defense system. GSH and other thiols, which are components of the intracellular antioxidant system, may not be adequate to repair this damage [37]. Statins have been reported to block the generation of reactive oxygen and nitrogen species, remove harmful free radicals, and boost the synthesis of vascular endothelial nitric oxide [56]. Accordingly, Pita was found to reestablish the oxidant-antioxidant equilibrium in the renal tissue.

Importantly, the effects of miR-93/AKT/mTOR signaling pathways and the link to Pita in the pathophysiology of renal tissue injury have never been elucidated *in vivo* through the context of xenobiotic toxicity.

MicroRNAs (miRNAs) play a significant role in various biological processes, including kidney injury and repair. One specific miRNA that has been studied in the context of kidney injury is miR-93. In addition, PTEN (phosphatase and tensin homolog) is a critical protein involved in regulating cell growth and survival [57]. Studies have shown that miR-93 can target PTEN, leading to its downregulation. This downregulation of PTEN, in turn, can contribute to kidney injury by promoting cell proliferation, survival, and inflammation. During kidney injury, there can be an upregulation of miR-93, leading to decreased PTEN levels and subsequent alterations in cell mir-93/AKT/mTOR signaling pathways. These changes may impact the overall injury and repair processes in the kidney [58]. It is worth noting that the study of miRNAs in kidney injury is an active area of research, and ongoing investigations continue to shed light on their specific roles and potential therapeutic implications. Our findings are consistent with the data presented, showing that miR-93 is linked to the activation of the AKT/mTOR pathway, resulting in kidney injury. On the contrary, the administration of Pita treatment in the current study successfully halted the miR-93 level, leading to the upregulation of the PTEN level. This, in turn, suppressed the AKT/mTOR pathway in the kidney, leading to the improvement and resolution of kidney injury.

A crucial transcription factor known as Stat3 controls cytokine-dependent inflammation and immunity [59, 60]. Cytokine receptors stimulate the Janus kinase (JAK)-Stats signaling cascade and phosphorylate Stat3. As a result, Stat3 either pairs up with other Stat proteins to form stable homodimers or heterodimers. These activated Stats then react to cytokines, creating a feed-forward inflammation loop within the affected microenvironment [61]. Studies using experimental models of kidney diseases have examined the role of Stat3 in terms of pathophysiology and the protective effects of Stat3 inhibition [62]. Stat3 is the target of miR-93 [63]. As shown by the decreased Stat3 expression generated by TAA in the renal tissue, Pita was able to lessen Stat3 activation in this case, protecting against renal injury. The observed results are consistent with the investigation of Al-Shabrawey et al. [64], who demonstrated that simvastatin could protect against the early signs of diabetic retinopathy by preventing NADPH oxidase-mediated activation of Stat3.

Further research is needed utilizing genetic manipulation of miR-93 to conclusively demonstrate its direct interactions with target genes like PTEN and precise impacts on Akt/mTOR signaling in this context. While beyond the scope of the current study, such molecular examination through gain and loss of function experiments represents an important future direction to complement our findings.

## 5. Conclusions

In this study, we demonstrate for the first time that pitavastatin attenuates xenobiotic-induced kidney injury in rats by regulating the miR-93/PTEN/Akt/mTOR signaling pathway. Specifically, we show that thioacetamide causes marked impairment in renal function and histopathological damage associated with oxidative stress, inflammation, and apoptosis. Pitavastatin treatment mitigated these harmful effects and preserved normal kidney structure and function. Mechanistically, our central finding is that the renoprotective impact of pitavastatin involves the suppression of miR-93 expression and subsequent activation of PTEN. This shifts the balance of Akt/mTOR signaling to ameliorate molecular processes driving kidney toxicity. Taken together, these novel results substantially advance understanding of the pleiotropic benefits of statins in the kidney by defining a specific molecular cascade—the miR-93/PTEN/Akt/mTOR axis—underlying their protective capacities.

Overall, this research highlights the therapeutic potential of pitavastatin in contexts of toxicant-mediated oxidative injury and inflammation in the kidney. Our work suggests that the modulation of miR-93 and associated signaling could be a promising pharmacological approach to attenuate the progression of acute kidney disease. These findings merit further investigation of the clinical applicability of statins to preserve renal function in patient populations at high risk of kidney damage due to xenobiotic exposures or conditions causing oxidative stress.

## Data Availability

The data used to support the findings of this study are included within the article.
